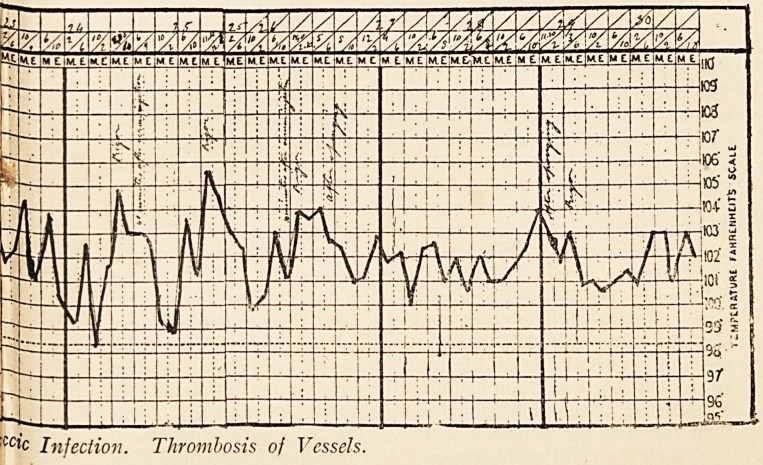# Cases Illustrating the More Unusual Complications of Pneumonia

**Published:** 1907-06

**Authors:** J. Michell Clarke

**Affiliations:** Professor of Medicine, University College, Bristol; Physician to the Bristol General Hospital.


### CASES ILLUSTRATING THE MORE UNUSUAL COMPLICATIONS OF PNEUMONIA.

BY

J. MICHELL CLARKE, M.A., M.D. Cantab., F.R.C.P.,

*Professor of Medicine, University College, Bristol;*
*Physician to the Bristol General Hospital.*

THE following cases illustrate some of the rarer complications met with in pneumonia. The occurrence of such complications forms one of the strongest arguments in support of the modern conception of pneumonia as a general infection. These instances occurred in a consecutive series of 126 cases of pneumonia. They comprise **peritonitis** (2); **thrombosis of vessels** (3), in one of which there was also general pneumococcic infection; **endocarditis** (2); **nephritis** (1); **arthritis** (1). In the same series there were also three cases of delayed or imperfect resolution of the consolidated lung, and three cases of empyema, neither of which I have thought it worth while to report. With regard to cases of imperfect or absent resolution, it may be remarked, however, that if an example of this condition comes under observation for the first time at some considerable interval after the original illness, valuable aid is given in diagnosis by the common circumstance that the consolidated area corresponds exactly to the superficial delimitation of one lobe of a lung on the surface of the chest.

There is also a point of importance in empyemata following pneumonia, that is, that at an early stage firm adhesions may unite the visceral and parietal pleuræ along the septa between the lobes of the lung, and thus on the right side there may be a collection of pus over the lower lobe entirely shut off by adhesions along the interlobar septum from another smaller collection over the middle lobe. This happened in one of the above cases of empyema, in which after the pus had been evacuated from an empyema over the right lower lobe it was found necessary to do a further operation for a small collection of pus exactly corresponding to the superficial extent of the middle lobe, and completely shut off from the first one.

**Pneumococcic Peritonitis.**—Peritonitis is a rare complication of pneumonia, although, as is well known, severe pain in the upper part of the abdomen accompanied by great diminution in the respiratory movements in the same situation is common, and occasionally leads to errors in diagnosis. Though peritonitis is said to occur in pneumonia by direct extension through the diaphragm, it would seem more generally the result of a general pneumococcic infection, as appears likely in the following instances, which occurred in two sisters.

Winifred B., æt. 9, admitted February 7th. The patient when æt. 3 had several attacks of convulsions, but with this exception, although never robust, she had had no serious illness. On January 15th she went to school apparently in good health, but returned midday with a pain in her stomach, and vomited. The next day she was delirious, but this delirium soon passed off. During the next fortnight she suffered from severe paroxysmal pain in the abdomen, relieved by rubbing it. For the first week she was repeatedly sick, and the abdomen swelled and became hard. She had a troublesome, painful cough, the tongue was thickly furred, and an eruption of labial herpes appeared.

On February 1st the skin gave way over the umbilicus, and about two quarts of thin, greenish-yellow fluid was discharged, with great relief to the pain. Since that day there had been a slight discharge from the umbilicus. The bowels were moved regularly throughout. On admission the child was thin and emaciated, with the remains of labial herpes about the mouth.

On admission, February 7th, the chest was small and badly formed. There was some dulness over the base of the right lung, with coarse râles in the axillary region, and over the left base bronchial breathing and some small crepitations. The heart was normal. The abdomen was distended, its walls rigid, and there was a feeling of resistance about the abdomen generally. It moved on respiration. There was a sinus, discharging pus, at the umbilicus. There was dulness over the front of the abdomen, but the flanks were resonant and there was no evidence of free fluid. The temperature was hectic, varying between normal in the morning and 101° at night.

The general appearance of the child and the physical signs in the abdomen suggested abdominal tuberculosis ; examination of the pus from the sinus, however, showed no tubercle bacilli, but the pneumococcus was obtained in pure culture.

The blood gave erythrocytes 2,650,000, white corpuscles 15,000 to c.mm. A small incision was made just below the umbilicus, and a large cavity, shut off from the rest of the peritoneum, was discovered between the omentum and the anterior abdominal wall, from which about two pints of pus were evacuated. After the operation the temperature fell to normal, the sinus gradually closed up, and she left the hospital quite well five weeks later.

On March 2nd Bessie B., æt. 4, the sister of the above patient, was admitted, with the history that on January 24th, three days after her sister was taken ill, she developed a bad cold, with fits of shivering and several attacks of vomiting. During the next two weeks she was very ill with high fever, a bad cough, and pains in the chest and abdomen. The pains in the abdomen were paroxysmal, at times very severe, and were relieved by poultices. There was diarrhœa at first, but the bowels were afterwards moved regularly. The vomiting ceased after the first week. The abdomen became swollen, distended, and hard, and as the child did not get better, she was brought to the hospital.

On admission the child was pale and emaciated. There were a few scattered râles at the bases of the lungs, otherwise there were no abnormal physical signs in the chest. The pulse was 120; respiration 30; temperature 99.5°. The abdomen was much distended, measuring 22½ inches at umbilicus. It was dull all over, except in the epigastric region. There was a distinct fluid thrill. The skin around the umbilicus was red and dusky. The abdomen moved very little with respiration.

The day after admission Mr. Morton made a small incision between the umbilicus and xiphoid cartilage, and evacuated a large quantity of greenish yellow, fairly thick pus. On examination the organisms obtained gave the cultural characteristics of the pneumococcus. The after history of the case was uneventful, the discharge rapidly diminished, and the patient left the hospital quite well twenty-five days later.

The interesting point is the occurrence of the same complication, and that an uncommon one, in two sisters, and also, in the first case, the close resemblance clinically to abdominal tuberculosis, at once cleared up by the discovery ot the pneumococcus in the pus.

**Cases of Thrombosis of Vessels.**—Thrombosis is a rare complication of pneumonia. In 27 out of 32 cases collected by Steiner the time of its appearance was during convalescence. According to Osler, it nearly always occurs in the femoral veins, and is still more uncommon in arteries than in veins.

*Thrombosis of Left Superficial Femoral Vein.* The patient was a strong, healthy collier, of temperate habits. He had a severe chill on December 24th, and on the 25th a rigor with pain in the left side of the chest, and was admitted into hospital on 26th with the signs of lobar pneumonia at the left base. The course of the pneumonia was uneventful, and terminated by crisis on December 31st. The heart's action was well sustained throughout; there were no murmurs; the pulse was good, varying from 80—96. A leucocyte count on the fifth day gave 45,000 to c.mm. The urine was normal. On the thirteenth day, when convalescence was apparently proceeding normally, the temperature, which had been normal for five days, rose to 99.5°, and he complained of pain in the left groin and calf, and the left thigh and leg swelled with great rapidity. The next day a hard, tender, band-like swelling could be felt along the course of the left superficial femoral vein, and a swollen vein on the calf leading up to this. There was no further constitutional disturbance, and no sign of any heart affection. Under the usual treatment pain and swelling gradually subsided, and by the end of January all evidence of the thrombosis had disappeared. He left the hospital well on February 3rd.

The most notable feature of the case is the rapid onset, and the equally rapid clearing up of the lesion, in marked contrast with the tedious course of most cases of thrombosis of the femoral vein. The cause was obscure; there was no general infection, and no affection of the heart, whilst the well-marked leucocytosis in a moderately severe attack of pneumonia showed that the patient's resistance was good.

*Thrombosis of Cerebral Vessels.* J. H., æt. 25, had always enjoyed good health, but was said to have had a slight discharge from the right ear before present attack. It was doubtful, however, whether this discharge was more than a little wax. He had felt ill for a week before, being obliged to give up work on October 29th. He was first seen by Mr. E. H. C. Pauli on October 31st, who found that his temperature was 102°, and that there was pneumonia of the lower lobe of the right lung. The crisis occurred on November 1st, and he was apparently doing well, when on November 3rd he had two slight rigors, an attack of general epileptiform convulsions, and his temperature, previously normal, rose to 101°. He vomited several times, and complained of headache. The temperature remained about 102°; there was no paralysis of any muscles, no recurrence of rigors, nor fresh symptoms, but on November 4th he became drowsy, and this gradually deepened into coma.

On November 6th, when I saw him, he was in a condition of moderately deep coma; he did not speak, but opened his eyes when shouted at. The pulse was 120; the respiration 32, irregular and often sighing. There was deficient resonance over the base of the right lung, and *redux* crepitant râles were heard here. The cardiac apex beat and area of dulness were normal, the pulmonary second sound accentuated and the first sound at the apex indistinct. Abdominal organs normal. The pupils acted to light, and were of moderate size, the right larger than the left. There was no localised paralysis; he could move his limbs; the arms were flaccid, the legs a little rigid at the knees. There was no rigidity of the neck muscles. The knee-jerks were present, not increased; the plantar reflexes were extensor in character; there was no ankle-clonus.

He could not swallow on this day; and there had been incontinence of urine and fæces for two days. The right ear contained wax, so that the drum could not be seen. The outline of the optic discs appeared a little blurred, but they were not hyperæmic, and there was no definite optic neuritis. On making a lumbar puncture, about 1½ ounces of perfectly clear, limpid fluid without any deposit were withdrawn.

In view of the difficulty of diagnosis in this case, the result of the lumbar puncture was especially important, as it enabled one to exclude meningitis, and probably intra-cranial abscess, a possible complication in view of the uncertain history of discharge from the ear. Thrombosis of cerebral veins was thought to be the most probable diagnosis.

The patient died the same evening. An examination of the brain was made at his home under considerable difficulties. Unfortunately, the result of this examination was negative, as the exact cause of the cerebral symptoms was not made out, but it was valuable as proving the absence of meningitis and intra-cranial abscess. Thrombosis of cerebral vessels is not always easily recognised, especially under the conditions in which the examination of the brain was made in this case ; and I think that this was the probable cause of death.

Apart from meningitis, paralysis of cerebral origin, generally hemiplegic, but sometimes monoplegic, occasionally occurs in pneumonia, either early in the disease or during convalescence.

In some of these cases of hemiplegia no gross lesion is found after death, and they are often attributed to the effects of toxins. In those cases in which there is a *post-mortem* lesion, it is generally softening from embolism or thrombosis. Probably in some cases reported as without lesion a patch of recent softening has escaped notice, which, if it is small, is quite possible.

*General Pneumococcic Infection—Thrombosis of Vessels.* The patient, E. C., was a horse-driver, æt. 23, who had never had a day's illness previously. The illness began with pain in the head on May 5th, followed on 6th by purulent discharge from the left ear, and on the 7th by severe pain in the left side of the chest, which interfered with respiration, and by vomiting. He was admitted to hospital on May 8th with well-marked signs of pneumonia of the lower lobe of the left lung. The heart was normal, the area of dulness not increased, and the pulmonary second sound well accentuated. Pulse 80 ; respiration rate 60 ; temperature 102.5° ; leucocytes 18,750 to c.mm. Urine contained no sugar or albumin. Chlorides greatly deficient. He went on fairly well until the eighth day, when he had a rigor, and his temperature rose to 105°. It was noticed that his neck was swollen, and that there was thrombosis of the left median basilic vein extending into the basilic and median cephalic veins, the left forearm being swollen. The leucocyte count was now 25,700 to c.mm. ; erythrocytes 4,000,000 ; hæmoglobin 90 per cent.

The following three days the temperature was normal, and the patient felt much better. On the twelfth day the temperature suddenly rose again to 105°. On this day a culture made from blood taken from the right arm showed a pure growth of pneumococci. During the next few days the patient felt better, in spite of the high range of temperature. Further, his tongue was red and dry, and his pulse, which had previously been about 68—72, now varied from 116—124. Leucocytes 21,800 to c.mm. The physical signs at the left base indicated that the consolidation was slowly clearing up in spite of the grave constitutional symptoms.

On the twenty-second day of the illness 5 cc. of antipneumococcic serum were injected. On the previous day he had had another rigor, and rise of temperature to 105°. The leucocyte count was now 19,375 to c.mm. A trace of albumin had appeared in the urine, which also gave a distinct band of urobilin.

On the twenty-fourth day, as the fever still continued high and the rigors recurred daily, a second injection of 5 cc. serum was given, with no appreciable effect, unless a fall of temperature from 103° to 101° be so reckoned.

There had been no extension of the thrombosis in the veins of the left arm, which remained limited to the veins in which it first appeared. No other thrombosed veins were detected elsewhere. There was no enlargement of the spleen that could be detected clinically at any period of the illness. The physical signs in the lungs remained stationary, the consolidation only partly clearing up.

On the twenty-eighth day of the disease the temperature was lower, at about 102°, but he was wandering in mind, looked haggard and emaciated, was sweating profusely, had partial incontinence of urine, and was obviously sinking. He died two days later.

The results of the *post-mortem* examination confirmed the diagnosis made during life. The thrombosis here was undoubtedly part of a severe general pneumococcic infection, and the gravity of the case was due to this latter condition, and not to the thrombosis, which could only be considered an incident in the illness, and not contributing to the lethal result.

**Endocarditis.**—*Double Pneumonia.* Maurice Y., æt. 19, engine cleaner. There was no history of any previous illness. For a few weeks the patient had suffered from an obstinate cold in the head, when on October 5th he was taken with pain in the right side of the chest, cough, fever and profuse sweats. He went to bed, and the next day the symptoms had increased, and he coughed up some rusty, tenacious sputum.

He was admitted on October 9th with well-marked signs of consolidation of the lower lobe of the right lung, and there was also dulness and bronchial breathing at the left base. The heart's apex was in the normal position, and the area of cardiac dulness normal. No murmur was heard. The temperature was 103° ; pulse 90 ; respiration 30, shallow and irregular.

On October 10th the signs at the left base had increased, and now indicated extensive consolidation of the lower lobe of the left lung. A pleuritic rub was audible all over this lobe, and there was also well-marked pleuro-pericardial friction. The temperature was 102° on this and the following day, October 11th, when his pulse was 130, respiration 50. No cardiac murmur could be heard, possibly on account of the loud friction sounds which were present throughout. On this day he became suddenly worse after a violent fit of coughing, was deeply cyanosed, and died of heart failure.

*Post-mortem.*—There was extensive acute pleurisy over the whole of both lungs. Nearly the whole of the right lung was in a state of red, passing in places into grey, consolidation, and the lower lobe of the left showed red hepatisation. The heart cavities were dilated, especially on the right side. On the mitral valves were some old granulations, and in addition on these and on the edge of the valve segments were numerous recent small granulations. Scrapings of the cut surface of the consolidated lung showed numbers of pneumococci, which were also obtained from the recent granulations on the mitral valve. The other cardiac valves were healthy.

Endocarditis is not a common complication of pneumonia. In the form of which the preceding case is an example it often goes unrecognised. In this case there was no evidence from the previous history or symptoms of the old existing lesion of mitral valve, and, further, there were no signs of the acute endocarditis supervening on this old lesion of the valve during the fatal illness. Any mitral murmur during this illness would have been effectually concealed by the loud friction sounds. The low temperature throughout such a severe attack of pneumonia is an unfavourable symptom, indicating poor powers of resistance on the part of the patient. Endocarditis in pneumonia generally affects the left side of the heart, and the pneumococcus is usually the active agent. As to its frequency, Preble, from an exhaustive analysis of over 20,000 cases, gives it as 1 per cent. of all and 5 per cent. of fatal causes. Pneumococcic endocarditis is frequently accompanied by meningitis, so that cerebral symptoms may form the predominant feature of the illness. Both this and the following case illustrate the well-established fact that cardiac valves which are the seat of previous disease are especially liable to be attacked by the micro-organisms of any subsequent acute infection.

*Pneumococcic (Ulcerative) Endocarditis.*—Charles M., æt. 47, a clerk, had scarlet fever as a child, and at the age of 22 was told by a medical man that he had heart disease. Except for occasional attacks of palpitation he remained well, and was able to do his work until April, 1900, when he was laid up with fever, cough, and pain in the right side, and was treated for a " liver attack." After this he got about again, but felt weak, and six weeks before admission took to bed on account of increasing weakness. He had a rise of temperature every evening, with profuse sweats between 3 and 6 a.m. There were no other symptoms, except a slight attack of hæmaturia three weeks before admission, and a rash consisting of bright red spots over the legs.

On admission he looked ill, and was anæmic and sallow. The temperature was 102° ; the pulse 96, collapsing ; respiration 26. There was a fading purpuric rash on the legs.

On examination the breath sounds were harsh, the lungs otherwise normal. The heart was much enlarged, the apex beat being two inches outside the left nipple. There was a marked diastolic thrill at the apex, and a loud systolic murmur there. At the base there was also a rough systolic murmur, followed by a long soft diastolic murmur conducted down the sternum. All the visible arteries showed marked pulsation, and capillary pulsation was also observed. The urine contained a little blood and albumin. The liver was slightly enlarged. The spleen was distinctly enlarged, its tip being felt about two inches below the lower border of the ribs. There were no retinal hemorrhages, and the optic discs were normal.

The temperature chart shows the irregular course of the fever, and the high range to which it reached. There were profuse nocturnal sweats. The symptoms and physical signs made the diagnosis of malignant endocarditis obvious. Blood was withdrawn from the veins of the arm on three occasions, in order if possible to ascertain the cause of the infection, but no micro-organisms could be recovered from it. At this time the history of the beginning of the illness, when he was treated for "liver complaint," did not have the significance which it afterwards obtained in the light of the pathological findings. In the absence of evidence of the organism present, it was thought advisable to try anti-streptococcic serum. Three brands of serum were employed, one of them being a polyvalent anti-streptococcic serum, and were given in doses of from 10—30 cc. In all 480 cc. were injected, so that a thorough trial of the serum was made. Neither of them had any appreciable effect either for bad or good, except for occasional local redness, infiltration, and tenderness at the site of injection.

He grew steadily worse, and on October 15th slight general anasarca and ascites appeared. This gradually increased, and œdema of the ankles was marked on October 22nd.

On October 26th there was thrombosis of the superficial veins of the right calf, and a fresh petechial eruption appeared over the left thigh and knee. The albumin in the urine increased. About this date he became much weaker and delirious at night. The heart's action became weaker and very irregular. The murmurs persisted as at first.

On October 30th there were signs of a little fluid in the pleural cavities and congestion of the bases of the lungs. He died on November 4th.

*Post-mortem.*—There was a large infarct into the upper lobe of right lung. The lower lobe of the left lung was completely airless, dark red, solid, and granular on section. The heart was greatly enlarged; both ventricles much dilated. On the auricular surface and free borders of the tricuspid valve segments were large masses of fungating, soft granulations, which extended on to the chordæ tendineæ. The mitral valves were much thickened and contracted, but contained no granulations. The pulmonary valves were normal. Aortic valves were largely destroyed, one segment almost entirely, and covered by large masses of granulation tissue. Scrapings from the valves, from the spleen, and from the pulmonary lesions showed abundant pneumococci, which were also stained in situ in sections made of the granulation tissue on the cardiac valves. The kidneys showed subacute nephritis, and there were a few small hemorrhagic infarcts in the jejunum.

There was no ground for the employment of anti-streptococcic serum in this case beyond the fact that a streptococcus is the most common cause of malignant edocarditis, and in the failure of all attempts to determine the exact organism present, and in view of the grave character of the illness, it was thought right to act on the hypothesis that this organism might be present. It shows, however, the fallacy of employing any serum in the absence of exact evidence of the kind of organism causing an infective illness. Looking back in the light of the *post-mortem*, the "liver attack" of the preceding April was probably a mild attack of pneumonia, but the history was too inconclusive for us to be able to come to any such conclusion during life.

**Nephritis.**—E. P., æt. 10. The patient's mother was taken ill with pneumonia on May 13th, and her father on June 2nd. The patient's illness began on June 6th with pain in the side, cough, fever, sickness, and diarrhœa. She did not sleep in the same room with her parents. She was admitted to the hospital on June 13th. She had not passed any urine for forty-eight hours before admission, and what was passed just before this period of suppression was the colour of blood. She was delirious at night.

On admission the temperature was 103°, pulse rate 104, respiration 42. The tongue was thickly furred. On examining the chest, there was pneumonic consolidation of the lower lobe of the right lung. The heart's apex beat was in the fourth space, and the cardiac dulness extended half an inch over the right border of the sternum. There were no murmurs. There was no enlargement of either liver or spleen. A leucocyte count gave 28,900 to c.mm. A few ounces of urine were passed, which contained a large quantity of albumin, much blood, and blood and epithelial casts.

On June 15th—16th (ninth and tenth days of disease) she was delirious, with a temperature rising to 104°, pulse 112, respiration 44. The abdomen was swollen and tense; the urine showed the same characters as above. Pallor of face and puffiness of lower eyelids were marked.

On the eleventh and twelfth days of the illness the crisis occurred, and on the following days the affected lung showed signs of resolution. Respiration fell to 24, and pulse to 72.

On June 20th (fourteenth day of illness) urine contained blood; epithelial, blood and leucocyte casts, with crystals of uric acid; there was still a large amount of albumin. Quantity passed, 32 ounces; urea in 24 hours, 26.5 grms. A leucocyte count on this day gave 28,000 to c.mm., and on June 26th 20,400 per c.mm.

Between June 20th and 26th the daily quantity of urine was only 16 to 20 ounces; on the latter date its sp. gr. was 1020, with no blood, no casts, and only a small quantity of albumin. The lung continued to undergo normal resolution, and by July 9th the mischief there had cleared up. On this day examination of the urine gave: quantity 24 ounces; sp. gr. 1020; urea daily excretion about 15 grms. on a milk diet with one egg; a trace of albumin only; uric acid and calcium oxalate crystals, epithelial cells from urinary passages, and a few granular casts.

On July 17th and 24th the face was puffy, and there was still a trace of albumin in the urine; otherwise the child seemed well. At the end of this month the urine was passed in normal amount with a sp. gr. of 1020; it contained no albumin and no deposit, and she was discharged well on August 5th.

No pneumococci were found at any time in the urinary deposit. Nephritis is a very rare consequence of pneumonia. This is the only case I have ever seen. I think there can be no doubt of cause and effect in this case. In so completely and quickly clearing up, it followed the usual course of nephritis in acute infective disease. The case is a good instance of *infective* pneumonia, and in the infective form; the disease is well known to be more severe, more apt to be a general infection, and therefore to lead to complications.

**Pneumonia, Empyema, Arthritis.**—Victor R., æt. 8, previously healthy. On April 16th, 1906, patient fell into a pool of water; on the 17th pains in the right hypochondrium and side of chest, followed on 18th by cough and pains in the left arm.

On 21st he was admitted into the hospital with a temperature of 102°, pulse 120, respiration 34. On examining the chest there was dulness, deficient air entry, a few moist crepitations, and increased vocal resonance over the base of the right lung. The other organs were normal. The left elbow-joint was swollen, red, and painful, and there was some fluctuation in the joint. On April 23rd there was a patch of bronchial breathing at the lower angle of the right scapula, otherwise the lung signs remained the same. The swelling of the elbow-joint also remained stationary; the pain was relieved by placing it upon a splint. The signs of

*Case of Pneumococcic Nephritis.*

*To show range of temperature in case of Pneumococcic (Malignant) Endocarditis.*

*Temperature Chart. General Pneumo*

an empyema having developed, on May 4th a portion of the eighth rib was excised, and a moderate quantity of thick yellow pus evacuated. The pus contained numbers of pneumococci. At the same time two incisions were made into the elbow-joint, but no pus found. On May 14th there was no pain in the elbow, and the swelling had much diminished, but the joint was stiff, with very little movement of flexion or extension, the former not beyond a right angle.

On May 17th the condition of the left elbow-joint still suggested the presence of pus, but none was found. The muscles of the arm were now much wasted, more especially the triceps. On the next day massage of the limb and gentle passive movements of the joint were begun. A curious feature of the case was the appearance of four or five large pustules on the left ankle and foot, which gradually died away, to be succeeded by a fresh crop on June 8th, which lasted until June 20th.

Another feature was that, although the empyema was opened and drained freely, and did well, the discharge gradually lessening, and there being no evidence whatever of retained pus, the temperature remained of a hectic type, ranging between 99° and 102°, for a fortnight after the operation before it returned to normal.

For the rest it is sufficient to say that the empyema wound healed about the middle of June, leaving a very good result as regards expansion of the lung, and that the swelling and stiffness of the joint gradually improved, so that when he left the hospital in July all movements were perfect and the joint appeared normal.

[illegible]ccic *Infection. Thrombosis of Vessels.*

## Figures and Tables

**Figure f1:**
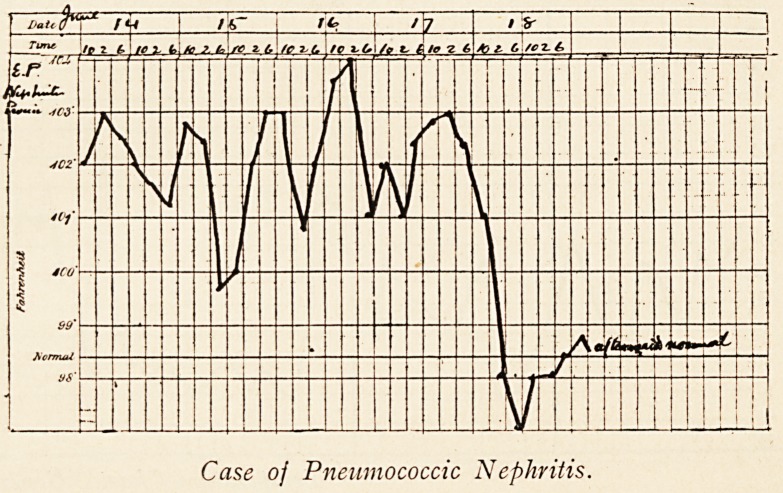


**Figure f2:**
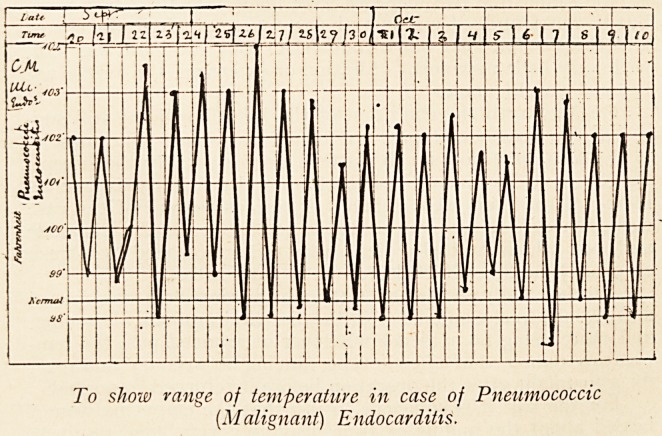


**Figure f3:**
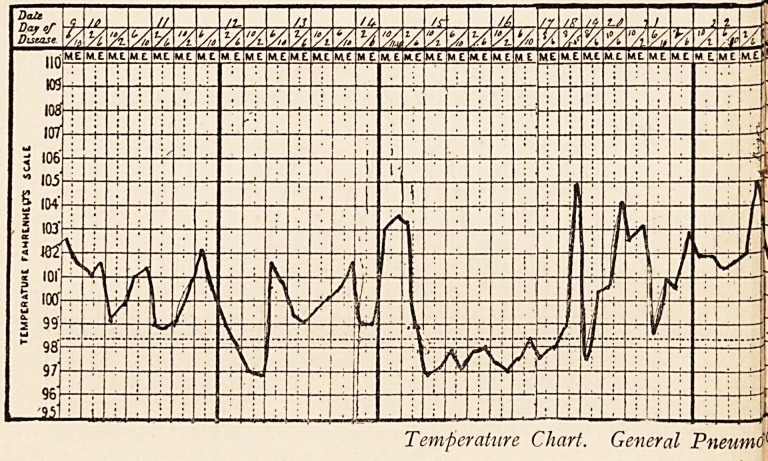


**Figure f4:**